# Stability of SiN_x_ Prepared by Plasma-Enhanced Chemical Vapor Deposition at Low Temperature

**DOI:** 10.3390/nano11123363

**Published:** 2021-12-11

**Authors:** Chi Zhang, Majiaqi Wu, Pengchang Wang, Maoliang Jian, Jianhua Zhang, Lianqiao Yang

**Affiliations:** Key Laboratory of Advanced Display and System Applications, Ministry of Education, Shanghai University, Yanchang Road 149, Shanghai 200072, China; zhangchi303145@163.com (C.Z.); wumjq@shu.edu.cn (M.W.); wangpc@shu.edu.cn (P.W.); smujml@163.com (M.J.); jhzhang@shu.edu.cn (J.Z.)

**Keywords:** SiN_x_, plasma-enhanced chemical vapor deposition, physical stability, thin-film encapsulation

## Abstract

In this paper, the environmental stability of silicon nitride (SiNx) films deposited at 80 °C by plasma-enhanced chemical vapor deposition was studied systematically. X-ray photoelectron spectroscopy and Fourier transform infrared reflection were used to analyze the element content and atomic bond structure of the amorphous SiN_x_ films. Variation of mechanical and optical properties were also evaluated. It is found that SiN_x_ deposited at low temperature is easily oxidized, especially at elevated temperature and moisture. The hardness and elastic modulus did not change significantly with the increase of oxidation. The changes of the surface morphology, transmittance, and fracture extensibility are negligible. Finally, it is determined that SiN_x_ films deposited at low-temperature with proper processing parameters are suitable for thin-film encapsulation of flexible devices.

## 1. Introduction

In recent years, due to the lightweight and bendable characteristics of flexible display, lots of interest has been attracted in flexible display technology and its wide application. Presently, display technology has gradually matured, but flexible screens have been difficult to launch in mass production so far. In addition to technical barriers such as integrated circuits, the biggest bottleneck lies in encapsulation [1,2]. Encapsulation is a crucial step for any electronic device because it would ultimately affect the reliability and maintenance of the devices. When manufacturing flexible display devices, glass is no longer used as a substrate and packaging cover. Components and adhesives need to have flexible features, and it is a huge challenge to ensure good packaging performance [3]. Thin-Film Encapsulation (TFE) adopts inorganic films for encapsulation, which can achieve flexibility and good optical transmittance at the same time. The inorganic materials are usually oxide or nitride of aluminum, silicon, or titanium [4,5,6]. However, S.-J. Yun et al. have found that the water vapor and oxygen isolation effect of SiO is worse than that of nitrides [7]. In addition, Al_2_O_3_ used for TFE usually use atomic layer deposition (ALD) deposition technology, this means that the film formation speed of Al_2_O_3_ would be significantly lower than plasma-enhanced chemical vapor deposition (PECVD) [1,7]. This obviously cannot meet the commercial requirements for preparing flexible device encapsulation films.

As we all know, silicon nitride (SiN_x_) films have great mechanical properties, such as the hardness, elastic modulus, and so on [8]. In addition, the SiN_x_ films also have efficient optical anti-reflection performance, high density and stability, and excellent water and oxygen isolation effect. These features are in line with the requirements of flexible devices for encapsulation. Therefore, SiN_x_ films are usually used for TFE [9,10,11] for the purpose of reducing the influence of water vapor and oxygen on the flexible display devices and increase the lifetime of the devices. SiN_x_ films can be deposited by various chemical techniques. However, high temperature, ultraviolet-ray (UV) radiation, plasma in the deposition process of organic and inorganic layers, chemical reactions between TFE materials and display film layers, and mechanical stress of TFE in the display area may cause the organic materials in the light-emitting layer to be damaged [12]. The most typical solution is that the substrate temperature during the packaging process must be less than or equal to 100 °C [13]. Therefore, the high-temperature process of traditional chemical vapor deposition (CVD) cannot meet the requirements. PECVD can achieve high-quality thin films at a low temperature under the electrical activation of plasma [7].

In addition, these films are inevitably exposed to various environmental conditions after deposition, including atmosphere, high temperature, moisture, and oxidizing media. The interactions at these conditions lead to changes in the properties of SiN_x_ films, which would also adversely affect the performance of the device. These interactions with the environment, mainly chemical processes in nature, all lead to the complexity of the film composition [14]. Z. Yongfa et al. have studied the composition, surface structure, thermal oxidation stability, and resistance to ion beam damage of SiN_x_ films prepared with PECVD [15]. In addition, a porous “fractal network” structure model was proposed to explain moisture penetration through micropores [16]. These reports focus on variation of the microstructure and elementary composition, but few researchers have explored whether the physical properties of SiN_x_ would change due to oxidation. Generally speaking, the penetration of water through TFE is caused by nanopores or defects, which are more serious at low deposition temperatures. Therefore, describing the atomic concentration, oxidation degree, and physical properties are necessary for studying the degradation process.

In general, there have been few studies on the stability of low-temperature SiN_x_ so far, especially the effect of oxidation on the macro-physical properties of low-temperature SiN_x_. In this paper, the environmental stability of SiN_x_ films deposited at 80 °C by PECVD was studied systematically. The atomic bond structures of non-stoichiometric SiN_x_ films were analyzed in depth [15]. In addition, the mechanical properties, transmittance, dielectric constant and surface morphology of the films were also evaluated. Finally, through systematic characterization, it is judged that the SiN_x_ films under low temperature with proper deposition process are suitable for film encapsulation.

## 2. Experimental

### 2.1. Preparation of the SiN_x_ Films

PECVD system (Japan, ULVAC, CME-200E) was used to deposit SiN_x_ films on four-inch (100) single-side polished crystal silicon wafers and 4 cm-long and 3 cm-wide transparent glass (USA, Corning, Eagle-XG). Silicon wafers and transparent glass need to be cleaned before deposition. They were sonicated with acetone, ethanol, and deionized water for 10 min, and then placed in a UV-ozone cleaning machine for 10 min. In addition, to measure the fracture strain of the SiN_x_ films, the SiN_x_ films were deposited on the prepared Polyimide (PI) films. The deposition gases were ultra-pure silane, ammonia, and nitrogen with a flow rate of 20, 80, and 270 sccm, respectively. The chamber pressure and radio frequency (RF) power were 100 Pa and 150 W, respectively. In this article, for the sake of exploring the characteristics and stability of low-temperature SiN_x_, the deposition temperature was set to be 80 °C.

Air atmosphere and accelerated aging environment (85 °C/85% RH) were used to study the oxidation and stability of low-temperature SiN_x_ over time in different environments. The temperature and humidity of the air environment were monitored and recorded in Table S1 (Supplementary Materials) in the Supplementary Materials for the purpose of comparing the oxidation conditions of the two environments more accurately.

### 2.2. Preparation of the PI Films

The PI slurry (Japan, Ube, U-Varnish S) was spin coated on cleaned glass substrates (USA, Corning, Eagle-XG) with two stage process: 700 rpm for 30 s and then 1500 rpm for 50 s. Then the samples were placed in a vacuum drying oven for 30 min at room temperature. Finally, the PI films were obtained after stepped annealing process: 120 °C for 30 min, 150 °C for 10 min, 180 °C for 1 h, and 450 °C for 5 min.

### 2.3. Characterization

In this experiment, an X-ray photoelectron spectrometer (XPS) (USA, Thermo Fisher Scientific, 250XI) was used for the semi-quantitative analysis of elements. Among them, the vacuum degree of the analysis chamber was 5 × 10^−10^ Pa, the excitation source adopted Al ka rays (hv = 1253.6 eV), and the C1s = 284.80 eV binding energy was used as the energy standard for charging correction [17]. Fourier transform infrared spectroscopy (FTIR) (USA, Thermo Fisher Scientific, IS50) was used to analyze the chemical bonding structure of SiN_x_. An infrared horizontal attenuation total reflection mode was used in the Fourier transform spectroscopy. The angle of incidence is 90. The oxidation condition and hydrogen (H) content variation can be estimated from the FTIR results [18].

In addition, the surface and the cross-section of the film were observed through SEM (Zeiss, merlin compact). Atomic Force Microscope (AFM) (Japan, Nanonavi, SPA-400 SPM) was used to characterize the surface morphology of these films. The transmittance of the SiN_x_ films deposited on glass was measured by a UV spectrophotometer (Japan, Techcomp, U-3900H). A nanoindenter (Germany, Hysitron, TI 950 Tribolndenter) was used to obtain elastic modulus and hardness, and all samples were tested at a maximum load of 1.5 mN and contact depths around 60 nm with the film thickness of about 851 nm.

For the sake of quantifying the fracture stress and strain of the SiN_x_ films, a real-time online observation system including a stretcher (USA, AMETEK, LS5) and a universal microscope (Japan, Nikon, SMZ745T) was established. The SiN_x_ film was fabricated on the prepared Polyimide (PI) film. The sample size is 10 mm wide and 50 mm long. The preload force is set to 1 N, and the preload speed is 0.5 mm/min. At the same time, for the convenience of observation, the elongation rate is set to 0.1 mm/min. In addition, when the load exceeds 30 N, the stretching stops.

## 3. Results and Discussions

**Figure 1:**
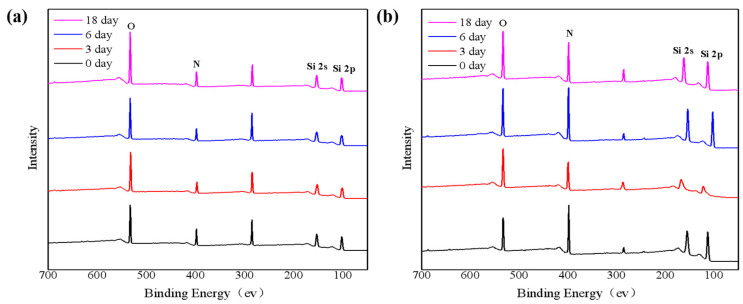
XPS analysis of (**a**) surface and (**b**) 200 nm inside of for SiN_x_ films oxidized at different times in the air.

Figure 1 shows the XPS spectra on the surface and 200 nm inside of the SiN_x_ films at room temperature, and the variation of 1s oxygen (O), 1s nitrogen (N), 1s carbon (C), 2s Si, and 2p Si XPS spectra with the time can be observed. Figure 1a shows the XPS spectra at the surface of the SiN_x_ films. The intensity of the O 1s in Figure 1a gradually increases with time, indicating that the film sample is gradually oxidizing [19]. The N 1s spectra approximately show constant high intensities through the thickness of the films, except for surfaces where the intensities are slightly reduced due to oxygen adsorption [17]. Most of the Si 2p spectra also show approximately constant peak intensity. However, the peaks are clearly distorted at the surface, indicating the coexistence of mixed bond states of silicon. Figure S1 (Supplementary Materials) shows the high-resolution Si 2p peak on the surface of the SiN_x_ film oxidized in the air. Figure S1b–d show a skewed peak. This peak is deconvoluted into two symmetrical peaks, corresponding to SiN_x_ and silicon oxide (SiO_x_), respectively. It shows that Si atoms are not only bond with N atoms, but some Si atoms begin to bond with O atoms. In addition, since the films are exposed to the air immediately after deposition, the films are easily contaminated with carbon, so relatively obvious C 1s peaks appear on the surface [20]. Although the films were tested by XPS in a vacuum analysis chamber, there could still be foreign carbon adsorbed in the machine. This may be one of the reasons for the nonmonotonic variation of the C 1s peak with time.

At the same time, for the sake of observing the oxidation inside the films, the films were etched with a depth of 200 nm in a high vacuum environment and the corresponding result are shown in Figure 1b. Compared with Figure 1a, the peak intensities of the N element are significantly increased, and the peak intensities of the O element are decreasing. This phenomenon shows that the degree of oxidation at 200 nm inside the film is far less than that of the surface. However, the O peak gradually increased with time, indicating that the degree of oxidation of the film at 200 nm was also increasing. It also shows that oxygen gradually penetrated from the surface downward over time.

**Figure 2:**
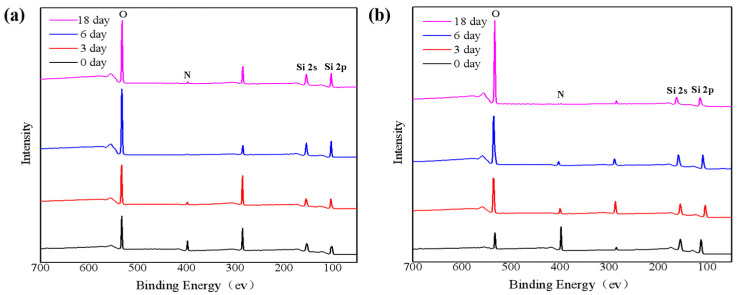
XPS analysis of (**a**) surface and (**b**) 200 nm inside of for SiN_x_ films oxidized at different times under 85 °C/85% RH.

Figure 2a shows the XPS spectra of the surface of the SiN_x_ films under 85 °C/85% RH. It is easy to observe that the intensities of the N spectrum decay significantly on the third day and almost disappear on the sixth day, indicating that SiN_x_ on the surface has been completely oxidized to SiO_x_ at this time. It can be concluded that the SiN_x_ film is more susceptible to oxidation under a higher temperature and humidity environment. The oxidation rate is significantly higher than that in the air. Compared with Figure 2b, the film sample etched at 200 nm can still show a faint N1s spectral peak on the 6th day, which shows that the SiN_x_ film has not been fully oxidized at 200 nm at this time, but it would be oxidized completely after 12 days. XPS analysis were performed on the as-deposited SiN_x_ film and the films placed in the air and under 85 °C/85% RH for 18 days, and the element content of these three samples was calculated. The results are shown in Table S2 (Supplementary Materials).

**Figure 3:**
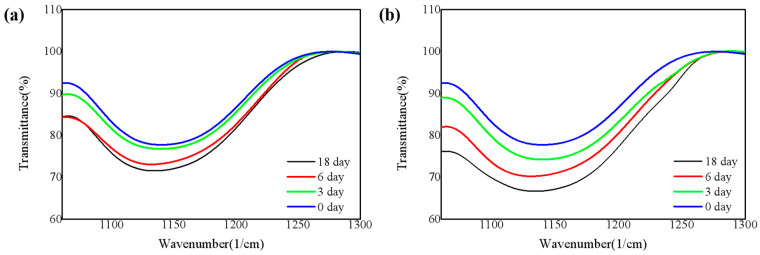
Infrared absorbance in the Si-O bond for four SiN_x_ films (**a**) in the air and (**b**) under 85 °C/85% RH.

To further study the chemical bonding structure, FTIR spectrum in the wavenumber range of 400–4000 cm^−1^ was obtained (Figure S2, Supplementary Materials). Figure 3 shows the silicon-oxygen (Si-O) bond absorption peaks of the four samples obtained from FTIR. These peaks correspond to the tensile vibration mode of the Si-O bond [15]. As shown in Figure 3a, the concentration of the Si-O bond gradually increases. Figure 3b is the same. In addition, by comparing the Si-O bond on the 18th day of the two environments, it is easy to see that the concentration of the Si-O bond placed under 85 °C/85% RH is significantly higher than that exposed to the air. It verifies that the samples are more likely to be oxidized at elevated temperature and moisture atmosphere, which is also consistent with the XPS analysis of the films.

The bonding method of H was analyzed through FTIR, to study the influence of dangling bonds-H bonds on the oxidation of SiN_x_ films, which cannot be ignored in low-temperature SiN_x_. Evidence for a large amount of bonded hydrogen is that it exists in the absorption spectra of N-H and Si-H stretching frequencies at 3350 and 2160 cm^−1^, respectively. The source of H is undoubtedly the silane and ammonia reactants [21]. We infer that in the early stages of oxidation, most of the H bonds would break and cause slight surface oxidation. Therefore, the breaking process of the H bonds can also indirectly reflect the oxidation process [22].

**Figure 4:**
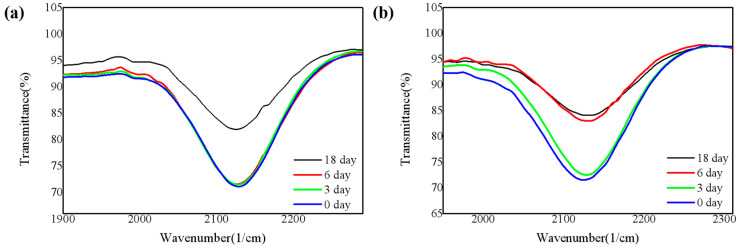
Infrared absorbance in the Si-H bond for four SiN_x_ films (**a**) in the air and (**b**) under 85 °C/85% RH.

**Figure 5:**
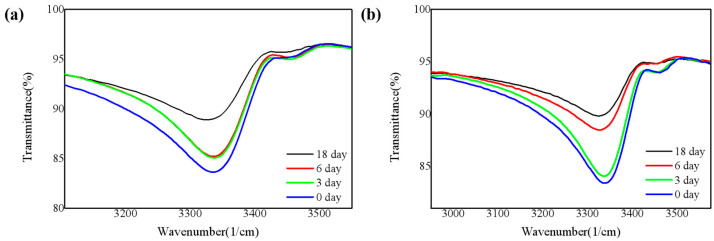
Infrared absorbance in the N-H bond for four SiN_x_ films (**a**) in the air and (**b**) under 85 °C/85% RH.

Figure 4 and Figure 5 show the absorption peaks corresponding to Si-H bonds and N-H bonds respectively [23]. As shown in Figure 4 and Figure 5, the deposited films contain both Si-H bonds and N-H bonds. The overall trend is that both Si-H bonds and N-H bonds decrease with time. In the case of higher humidity, the oxidation is more intense, and the H bonds are more likely to break to form Si-O bonds, so that the Si-H bonds and N-H bonds gradually decrease, and the Si-O bonds increase.

The biggest problem with the microstructure defects of the inorganic thin-film encapsulation layer is the high porosity at a low temperature. Due to the porous structure of the high-porosity films, water vapor can penetrate the nano-scale channels [24,25,26,27]. The surface and the cross-section of the film were observed through SEM. As shown in Figure S3a (Supplementary Materials), the surface of the film is flat, and the SiN_x_ film and the Si substrate have a very clear dividing line.

Figure S3b shows that the cross-sectional image of the SiN_x_ thin film of each thickness measured by SEM, showing thin-film structures without columnar structures that could be caused by the diffusion of defects such as grain boundaries.

To further study film flatness, the AFM analysis was performed to identify the surface topographies of the films after oxidation in the air environment and aging environment (Figures S4 and S5, Supplementary Materials). As shown in Figure S4, the surface of the SiN_x_ film is relatively flat, but there would be island-like deposits of about 3 nm [19,27]. Z. Chongyou pointed out that the island-like and cluster-like morphologies formed on the surface of the film would damage the surface flatness of the film [28]. Therefore, the root mean square (RMS) roughness over a scanned area of 2 × 2 nm of different barrier structures before and after oxidation was deduced. The results show that the roughness of the SiN_x_ films was basically maintained at about 1.3 nm. The flatness was high, and the surface morphology did not change significantly over time. Similarly, almost no change in the surface morphology of SiN_x_ films placed under 85 °C/85% RH was found as shown in Figure S5. It demonstrates that the surface morphology and flatness of the low-temperature SiN_x_ film did not change significantly due to oxidation.

**Figure 6:**
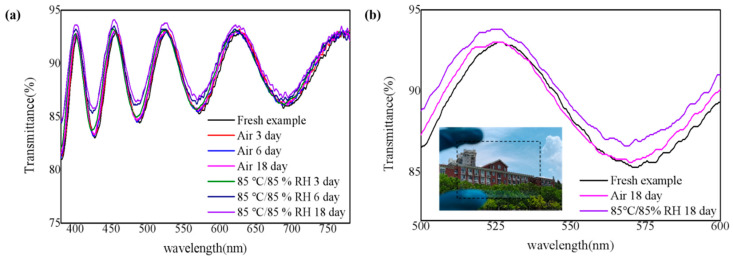
(**a**) The transmittance of SiN_x_ films in the 380–780 waveband and (**b**) transmittance of part SiN_x_ films in the 500–600 waveband, inset is the photo of SiN_x_ film on glass with film thickness of 851 nm.

Transmittance is a key factor as TFE film of flexible display, and the corresponding result is shown in Figure 6. Due to the interference effect of the upper and lower surfaces of the SiN_x_ films to the light, the transmittance changes in a sinusoidal fluctuation trend [1]. As shown in Figure 6a, as the oxidation time increases, the transmission performance of the SiN_x_ film hardly changes. Only in the 400–600 waveband, the transmittance of the film has a slight upward trend. Three samples were selected for comparison in the 500–600 waveband as shown in Figure 6b to better observe the changes in the permeability of the SiN_x_ film in the reference wavelength range. Inset graph of Figure 6b visually demonstrate the good transmittance of the SiN_x_ film deposited on glass. It is believed that when the ratio of nitrogen to oxygen decreases, the extinction coefficient gradually decreases, and the ability of the film to absorb light decreases, which leads to an increase in the transmittance of the film [29].

**Figure 7:**
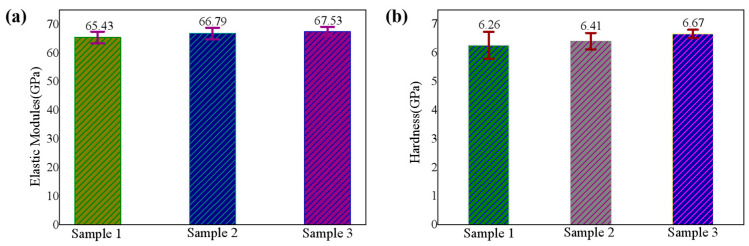
(**a**) Elastic modulus and (**b**) hardness of SiN_x_ films (sample 1 is as-deposited, sample 2 is exposed to the air and sample 3 is placed under 85 °C/85% RH for 18 days).

Hardness and elastic modulus are important indicators to judge the mechanical properties of the SiN_x_ film. Same as the traditional microhardness experiment, the nanoindentation experiment includes three stages of loading, staying, and unloading. Generally, elastic modulus and hardness of the film can be obtained by analyzing the loading and unloading curves. Three samples were selected for the study. Sample 1 is a fresh SiN_x_ film, sample 2 is exposed to the air for 18 days, and sample 3 is placed under 85 °C/85% RH for 18 days. Figure 7 shows the changes of elastic modulus and hardness of three film samples, respectively. The average value of elastic modulus changed from 65.43 to 67.53 GPa and the average value of hardness changed from 6.26 to 6.67 GPa. However, both the increase in the average values of elastic modulus and hardness is within one standard deviation of uncertainty. In other words, the elastic modulus and hardness did not change significantly during the oxidation process.

**Figure 8:**
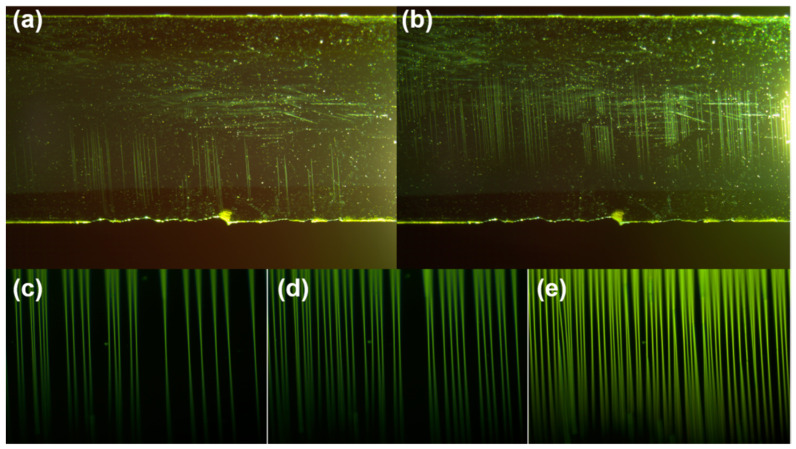
(**a**) Initial cracks, (**b**) perforated cracks of real-time monitoring image of cracks in SiN_x_ film and (**c**–**e**) at higher strain midpoint cracking begins and increases.

For the thin-film packaging of flexible devices, bending performance is particularly important. However, the fracture stress of the film in the bending state is not easily obtained by experiment. Some researchers have described the relationship between the fracture stress in the stretching state and the initial strain of the crack in the bending state for the coating [30]. The fracture strain of the films is closely related to the bending performance of the films. Bending and stretching would cause cracks in the inorganic film layer, and ultimately lead to a serious failure of the luminescent material of the internal protected devices. Through observation, it is found that the SiN_x_ films would have three stages of damage in the tensile experiment. As shown in Figure 8a, initial cracks began to appear in the first stage. The crack starts from one end of the film and starts to develop perpendicular to the loading direction under the initial strain of the crack. Figure 8b shows that the crack penetrates as the tensile force increases. Figure 8c–e shows that the second stage of film damage is midpoint cracking. New cracks generate between the two cracks and the crack density further increases. In the third stage, the film cracks appear delamination and saturation. No further cracks were generated at this stage, and the crack density reached the saturation value [8]. Due to the thinness of the PI substrate during this experiment, PI was broken before the crack density reach saturation.

**Figure 9:**
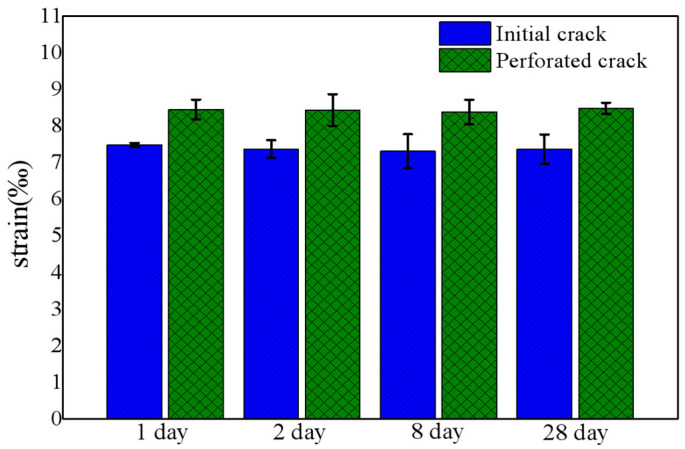
Fracture extensibility of SiN_x_ films at different time.

Figure 9 summarizes the fracture strain of the SiN_x_ films. All the strains when the initial cracks appear at about 7.2‰, while the perforated cracks remain around 8.6‰. This indicates that the oxidation of the SiN_x_ film does not reduce the bending resistance of the film, and the mechanical properties of the film are relatively stable.

## 4. Conclusions

The XPS analysis and FTIR analysis were used for the stability study of the low-temperature non-stoichiometric SiN_x_ film deposited by PECVD. It is found that as long as the SiN_x_ films are exposed to the air, they would be oxidized immediately. The O element slowly penetrates the films from the surface, and the longer the time, the greater the depth of oxidation inside the films. The films placed under 85 °C/85% RH were oxidized more severely. Similarly, the results of FTIR are in accordance with the XPS analysis which confirms that the oxidation of the film is greatly affected by moisture and temperature. In addition, the changes of elastic modulus, hardness, transmittance are not obvious as the degree of oxidation increases. The strain when the initial and through crack basically remain stable. Therefore, we can conclude that as the degree of oxidation increases, the element content and atomic bond structure of the SiN_x_ film have a certain change, but the mechanical and optical properties of the films are not affected significantly. The stability of low-temperature SiN_x_ is evident. Therefore, due to its extremely high stability, it is an ideal material for the thin-film packaging of flexible devices.

## Data Availability

The data generated during and/ or analyzed during the current study are available from the corresponding authors on reasonable request.

## Supplementary Materials

The following are available online at https://www.mdpi.com/article/10.3390/nano11123363/s1, Figure S1: (**a**) Si 2p spectral analysis of the surface of fresh SiN_x_ and Si 2p spectral analysis of surfaces of SiN_x_ of oxidized in the air for (**b**) 3 day and (**c**) 6 day and (**d**) 18 day, Figure S2: Full FTIR spectrum in the wavenumber range of 400–4000 cm^–1^ of SiN_x_, Figure S3: (**a**) Surface and (**b**) cross-sectional view of SiN_x_ taken by SEM, Figure S4: (**a**) surface of fresh SiN_x_ of AFM and surfaces of SiN_x_ of AFM oxidized in the air for (**b**) 3 day and (**c**) 6 day and (**d**) 18 day, and (**e**) three-dimensional image of fresh SiN_x_ of AFM and three-dimensional images of SiN_x_ of AFM oxidized in the air for (**f**) 3 day and (**g**) 6 day and (**h**) 18 day, Figure S5: (**a**) surface of fresh SiN_x_ of AFM and surfaces of SiN_x_ of AFM oxidized under 85 °C/85% RH for (**b**) 3 day and (**c**) 6 day and (**d**) 18 day and (**e**) three-dimensional image of fresh SiN_x_ of AFM, and three-dimensional images of SiN_x_ of AFM oxidized under 85 °C/85% RH for (**f**) 3 day and (**g**) 6 day and (**h**) 18 day, Table S1: The monitoring records of temperature and humidity under air atmosphere, Table S2: Element content of SiN_x_ films by XPS.
