# Supplementary material for: Stability of SiNx Prepared by Plasma-Enhanced Chemical Vapor Deposition at Low Temperature
Source: Nanomaterials (Basel). 2021 Dec 11;11(12):3363. doi: 10.3390/nano11123363 (PMC8706910; doi:10.3390/nano11123363)
Supplement: Supplementary file 1 [file nanomaterials-11-03363-s001.zip › nanomaterials-1455376-supplementary.pdf]

nanomaterials

Supplementary Materials

Stability of SiN_x Prepared by Plasma-Enhanced Chemical Vapor Deposition at Low Temperature

Chi Zhang, Majiaqi Wu, Pengchang Wang, Maoliang Jian, Jianhua Zhang and Lianqiao Yang *

Key Laboratory of Advanced Display and System Applications, Ministry of Education, Shanghai University, Yanchang Road 149, Shanghai 200072, China; zhangchi303145@163.com (C.Z.); wumjq@shu.edu.cn (M.W.); wangpc@shu.edu.cn (P.W.); smujml@163.com (M.J.); jhzhang@shu.edu.cn (J.Z.)
* Correspondence: yanglianqiao@i.shu.edu.cn

Table S1. The monitoring records of temperature and humidity under air atmosphere.

Days/Day	Temperature /°C(8 am)	Relative Humidity/%	Temperature /°C(5 pm)	Relative Humidity/%
1	20.1	39.7	20.8	41.8
2	21.9	37.4	22.6	39.6
3	22.6	43.2	22.7	43.9
4	22.9	44.2	23	46.8
5	22.3	39.3	22.9	41.3
6	22.3	36	22.8	37
7	22	32	22.3	30.6
8	21.3	26.2	21.9	28.5
9	21.4	26.5	23.2	28.3
10	21	26.4	21.5	29.9
11	21.4	34.1	21.8	34.8
12	21.9	29.7	21.9	30.2
13	21.9	29.4	22.2	33.5
14	21	28.5	22.1	28
15	22.1	27.8	22.2	30.2
16	21.9	34	22.8	40
17	22.5	39.8	23	37.5
18	23	37	22.2	32.5

Table S2. Element content of SiN_x films by XPS.

	The Surface				200 nm			
	N/%	O/%	Si/%	C/%	N/%	O/%	Si/%	C/%
As-deposited	10.69	24.11	29.03	36.17	24.25	13.92	44.9	16.93
In air for 18 days	13.88	23.99	30.39	31.74	21.76	16.99	39.59	21.66
Under 85°C/%RH for 18 days	2.7	41.27	26.43	29.6	5.25	43.26	30.99	20.5

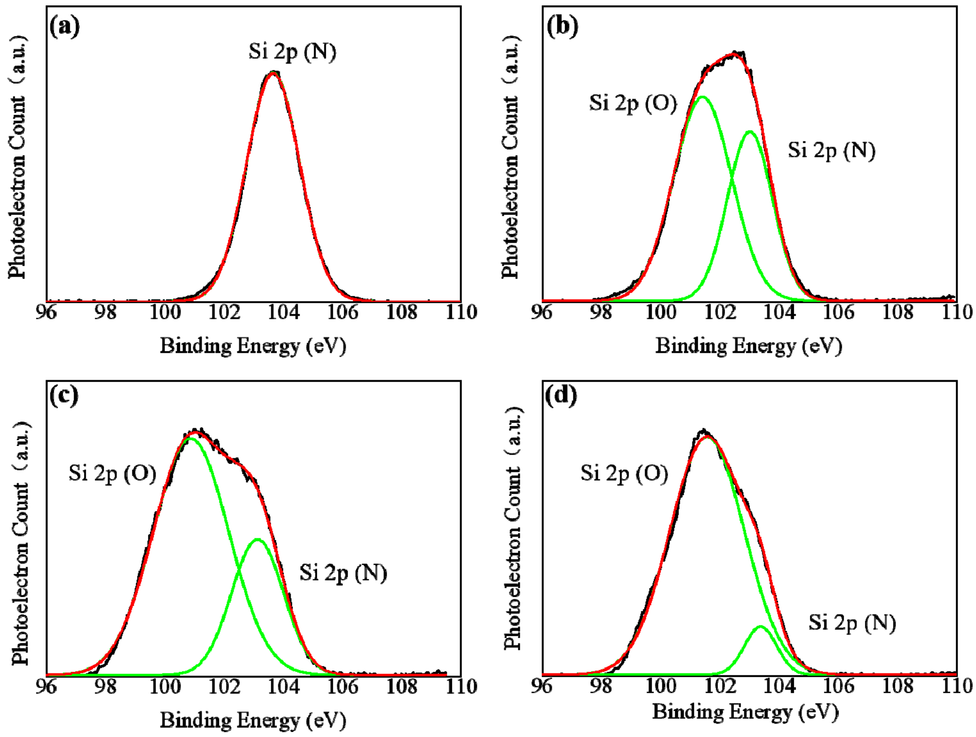

Figure S1. (**a**) Si 2p spectral analysis of the surface of fresh SiN_x and Si 2p spectral analysis of surfaces of SiN_x of oxidized in the air for (**b**) 3 day and (**c**) 6 day and (**d**) 18 day.

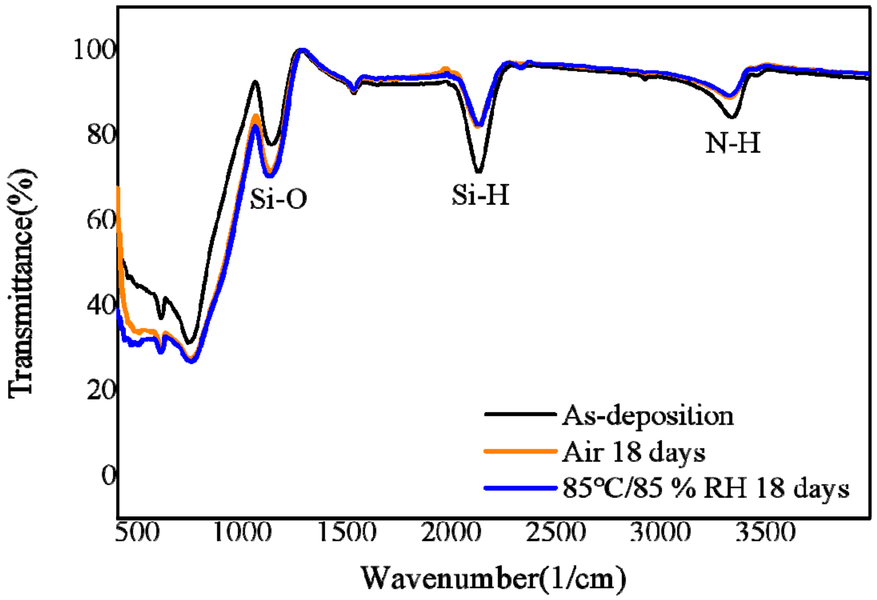

Figure S2. Full FTIR spectrum in the wavenumber range of 400–4000 cm^{-1} of SiN_x.

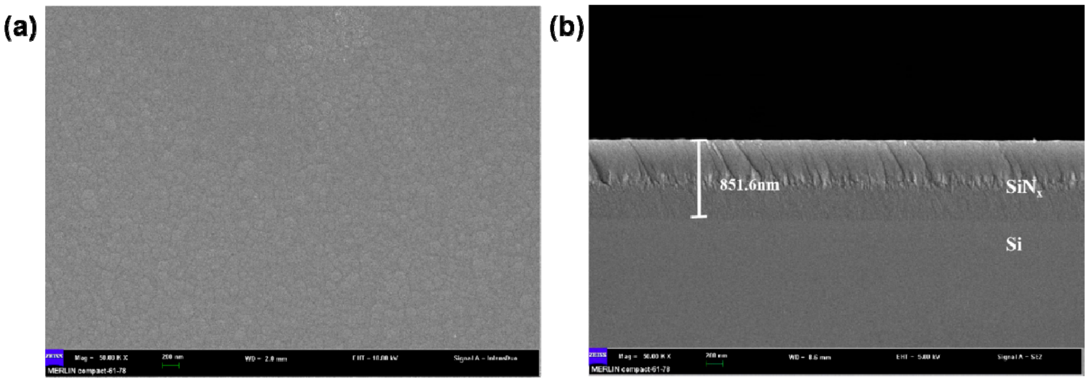

Figure S3. (**a**) Surface and (**b**) cross-sectional view of SiN_x taken by SEM.

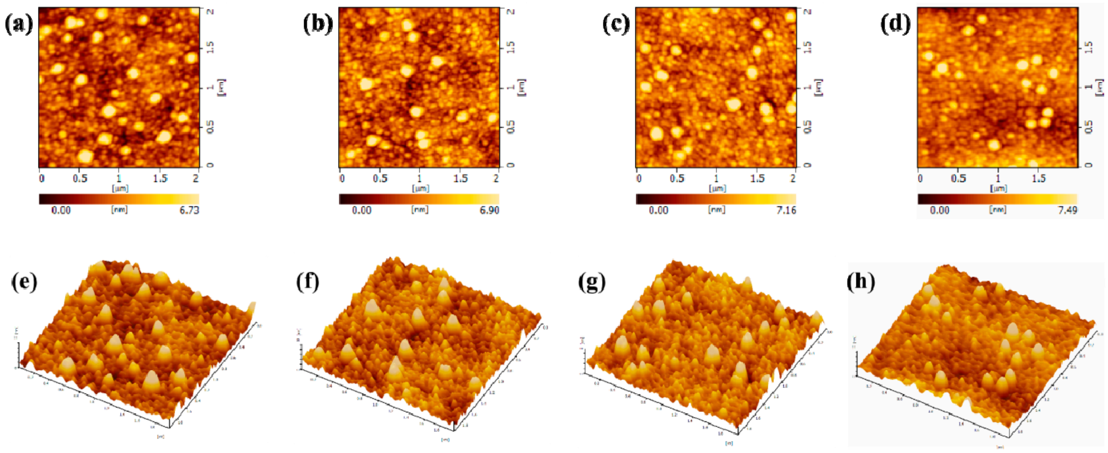

Figure S4. (**a**) surface of fresh SiN_x of AFM and surfaces of SiN_x of AFM oxidized in the air for (**b**) 3 day and (**c**) 6 day and (**d**) 18 day, and (**e**) three-dimensional image of fresh SiN_x of AFM and three-dimensional images of SiN_x of AFM oxidized in the air for (**f**) 3 day and (**g**) 6 day and (**h**) 18 day.

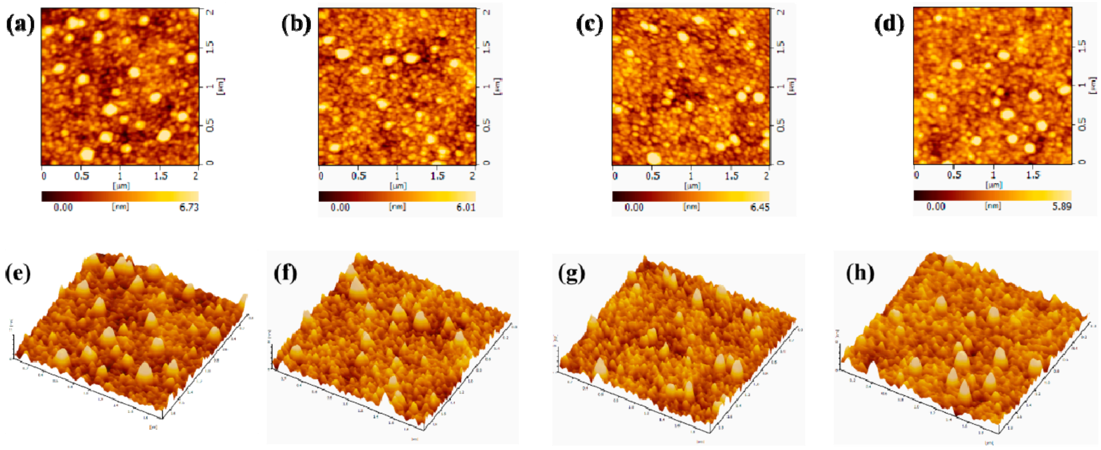

Figure S5. (**a**) surface of fresh SiN_x of AFM and surfaces of SiN_x of AFM oxidized under 85 °C/85 %RH for (**b**) 3 day and (**c**) 6 day (**d**) 18 day and (**e**) three-dimensional image of fresh SiN_x of AFM, and three-dimensional images of SiN_x of AFM oxidized under 85 °C/85 %RH for (**f**) 3 day and (**g**) 6 day and (**h**) 18 day.
